# A New Landscape for Systemic Pharmacotherapy of Recurrent Glioblastoma?

**DOI:** 10.3390/cancers12123775

**Published:** 2020-12-15

**Authors:** Giuseppe Lombardi, Ahmed Idbaih, Emilie Le Rhun, Matthias Preusser, Vittorina Zagonel, Pim French

**Affiliations:** 1 Department of Oncology, Oncology 1, Veneto Institute of Oncology IOV-IRCCS, via Gattamelata 54, 35128 Padua, Italy; vittorina.zagonel@iov.veneto.it; 2 Sorbonne Université, Inserm, CNRS, UMR S 1127, Institut du Cerveau, ICM, AP-HP, Hôpitaux Universitaires La Pitié Salpêtrière - Charles Foix, Service de Neurologie 2-Mazarin, F-75013 Paris, France; ahmed.idbaih@aphp.fr; 3 Department of Neurology, University Hospital and University of Zurich, Frauenklinikstrasse 26, 8091 Zurich, Switzerland; emilie.lerhun@usz.ch; 4 Department of Neurosurgery, University Hospital and University of Zurich, Frauenklinikstrasse 10, 8091 Zurich, Switzerland; 5 Division of Oncology, Department of Medicine I, Medical University of Vienna, Waehringer Guertel 18-20, 1090 Vienna, Austria; matthias.preusser@meduniwien.ac.at; 6 Department of Neurology, Erasmus University Medical Center, Doctor Molewaterplein 40, 3015 GD Rotterdam, The Netherlands; p.french@erasmusmc.nl

Glioblastoma is the most common and aggressive primary malignant brain tumor in adult patients. Despite multimodal treatment with maximal safe surgical resection followed by concurrent radiochemotherapy and adjuvant chemotherapy with temozolomide, the prognosis remains poor with a median survival of 1 year. The survival rate at 5 years is 5% and, theoretically, all glioblastomas relapse [1]. Once tumors progress, treatment options are limited, and the management of recurrent glioblastoma remains challenging. The standard of care for second-line treatment has not been defined; options include surgery, re-irradiation, systemic pharmacotherapy, and, in Europe, mostly lomustine [2,3]. The general and neurological health status of the patient, the molecular findings, the time to first recurrence, the pattern of recurrence, and previous treatment have to be considered. Nitrosoureas or temozolomide rechallenge show limited efficacy. Anti-vascular endothelial growth factor (VEGF) bevacizumab prolonged progression-free survival (PFS), but did not improve overall survival (OS) [4].

Because the treatment of recurrent glioblastoma remains a highly unmet clinical need, deeper mechanistic insight into the molecular changes present in these tumors is required. The enrollment of recurrent glioblastoma patients in clinical trials investigating new therapeutic options should be considered carefully whenever possible. In this Special Issue, we present some of the exciting updates on recurrent glioblastomas, which may help shape future treatment options.

In recent years, immunotherapy, especially the immune checkpoint inhibitors (ICIs) nivolumab, pembrolizumab, and durvalumab, showed durable improved outcomes in several types of tumors but not in the overall glioblastoma population [5]. Checkmate 143 was the first phase III trial investigating the efficacy and safety of nivolumab in recurrent glioblastoma patients [6]. A total of 369 cases were randomized to receive bevacizumab or nivolumab. The primary endpoint of overall survival was similar between the two arms: 9.8 and 10.0 months for nivolumab and bevacizumab, respectively. In this trial, the progression-free survival was even longer in the standard treatment arm: 1.5 months for nivolumab and 3.5 months for bevacizumab. In contrast to non-neurological cancers, no predictor of response to ICI was identified in recurrent glioblastoma patients [7]; mismatch repair deficiency and high tumor mutational burden did not correlate with the benefit of ICI in these patients in terms of overall survival [8]. The poor results of ICI in this tumor may be due to the high number of immunosuppressive CD68+ myeloid cells, the low presence of cytotoxic T lymphocytes in the tumor microenvironment, the poor quality of neoantigens, and the limited penetration of ICI within the brain [8].

Several clinical trials have evaluated the activity of molecular targeted therapies, mostly tyrosine kinase inhibitors (TKIs), which can block the biological mechanism implicated in cancer cell proliferation and angiogenesis. The BRAF protein is involved in the mitogen-activated protein kinase (MAPK) pathway, and the most frequent mutation in the BRAF gene results in V600E. The frequency of this mutation in glioblastoma is about 3%; in specific subgroups of glioblastoma, such as epithelioid glioblastoma, it can reach 50%. Vemurafenib, a potent BRAF V600E inhibitor, was analyzed in the VE-BASKET trial, reporting promising results. In this study [9], 24 patients with BRAF V600-mutant recurrent gliomas were enrolled, including six glioblastomas and five anaplastic astrocytomas: the median PFS was 5.3 months (95% CI, 1.8–12.9), the median OS was 11.9 months (95% CI, 8.3–40.1), and the objective response rate (ORR) was 9.1%. Notably, major activity was demonstrated with combined BRAF and MEK inhibition using dabrafenib plus trametinib, concurrently. The ROAR trial [10], a phase II basket study, analyzed this combination in many types of tumors including 39 recurrent high-grade gliomas with BRAF V600E mutation; the results were interesting, with an ORR of 27% (95% CI, 13.8–44.1), including one complete response and a disease control rate (DCR) of 57%.

Another important success in recurrent glioblastoma patients is attributed to larotrectinib, a selective TRK inhibitor. A study analyzed 18 cases with primary brain tumors, including six patients with recurrent glioblastoma, reporting a DCR of 100% [11]. Overall, these results highlight the potential use of these molecular targeted therapies in recurrent glioblastoma, despite the relatively low incidence in adult patients [12].

Regorafenib, another tyrosine kinase inhibitor of VEGFR1,2,3, TIE 2, PDGFR, FGFR, KIT, RAF-1, RET, and BRAF, was evaluated in the REGOMA trial, a randomized phase II study enrolling 119 recurrent glioblastoma patients [13]. In this Italian study, patients treated with regorafenib had a significantly longer overall survival of 7.4 months compared to 5.6 months with lomustine (HR 0.5, 95% CI, 0.33–0.75; *p* = 0.0009); the 6-month progression-free survival (6 m-PFS) rate and the ORR were also higher in the experimental arm (6m-PFS was 16.9% (95% CI, 8.7–27.5) in the regorafenib arm compared to 8.3% (95% CI, 3.1–17.0) in the lomustine group). Based on these results, regorafenib was included in the NCCN 2020 guidelines as a preferred regimen for recurrent glioblastoma patients, and the Italian Agency of Medicine (AIFA) also approved its use for Italian patients. Interestingly, two ancillary studies identified specific molecular alterations, such as phosphorylated acetyl-CoA carboxylase, as predictors of regorafenib efficacy [14,15]. At present, the Italian prospective and observational study REGOMA-Oss and the international phase III study GBM AGILE are ongoing to confirm and evaluate the benefit of regorafenib in recurrent and newly diagnosed glioblastoma patients. Of note, regorafenib can decrease the presence of immunosuppressive myeloid cells within the tumor; thus, it may increase the efficacy of ICIs. Based on this rationale, a new basket trial (Bayer 21136) will evaluate the combination of regorafenib plus nivolumab in many types of tumors, including recurrent glioblastoma.

The combination of TKIs and ICIs was recently studied in the LEAP-005 basket trial; this trial enrolled 31 recurrent glioblastoma patients treated with pembrolizumab plus lenvatinib, a tyrosine kinase inhibitor. It reported impressive results for recurrent glioblastoma with an ORR and DCR of 16.1% (95% CI, 5.5–33.7) and 58.1% (95% CI, 39.1–75.5), respectively.

Despite these encouraging results, many other studies analyzing targeted therapies, such as agents targeting c-MET, the PI3K–AKT–mTOR pathway, FGFR, and EGFR inhibitors, failed to improve outcomes in recurrent glioblastoma patients [5]. These poor results may also be due to molecular and genetic differences between tumors at diagnosis and at recurrence [16]; trials at tumor recurrence are usually based on molecular data from the primary tumor, with the potential loss of original mutations at relapse.

In conclusion, although the cornerstone of treatment in newly diagnosed glioblastoma remains radiochemotherapy with temozolomide, we are seeing some promising results in the setting of recurrent glioblastoma due to specific targeted therapies (i.e., BRAF inhibitors, TRK inhibitors, and regorafenib) alone or combined with immunotherapy in subpopulations of recurrent glioblastoma patients based on phase II clinical trials. The benefit of these new treatments needs further evaluation including: (i) larger phase III studies or trials based on new statistical approaches and (ii) companion tests examining predictors of response.
